# Study on the interactions between melamine-cored Schiff bases with cucurbit[*n*]urils of different sizes and its application in detecting silver ions

**DOI:** 10.3762/bjoc.17.204

**Published:** 2021-12-17

**Authors:** Jun-Xian Gou, Yang Luo, Xi-Nan Yang, Wei Zhang, Ji-Hong Lu, Zhu Tao, Xin Xiao

**Affiliations:** 1 Key Laboratory of Macrocyclic and Supramolecular Chemistry of Guizhou Province, Guizhou University, Guiyang 550025, China

**Keywords:** cucurbiturils, melamine, Schiff base, silver ion

## Abstract

Three different complexes, TMeQ[6]-TBT, Q[7]-TBT, and Q[8]-TBT are constructed by three different cucurbiturils and synthesized by guest melamine-cored Schiff bases (TBT) through outer-surface interaction and host–guest interactions. TBT forms a TMeQ[6]-TBT complex with TMeQ[6] through outer-surface interaction, while Q[7]-TBT and Q[8]-TBT form complexes with Q[7,8] through host–guest interactions. Among them, Q[7]-TBT is selected as a UV detector for the detection of silver ions (Ag^+^). This work makes full use of the characteristics of each cucurbituril and melamine-cored Schiff base to construct a series of complexes and these are applied to metal detection.

## Introduction

Schiff bases [1] are usually synthesized by the condensation of amines and active carbonyl compounds, endowing them both nitrogen and oxygen donor atoms [2–5]. Schiff bases are not only easy to coordinate with various transition metal ions to yield different metal-organic frameworks [6–11], but also can be used as analytical reagents for the detection of different organic and inorganic substances [12–14]. Among the various central molecules for the synthesis of Schiff bases, melamine (2,4,6-triamino-s-triazine) has attracted much attention due to its three-branched structure and excellent physical and chemical properties, which is commonly used in many applications including plastic dishes, the main raw material for formaldehyde resin, etc*.* [15].

**Scheme 1:**
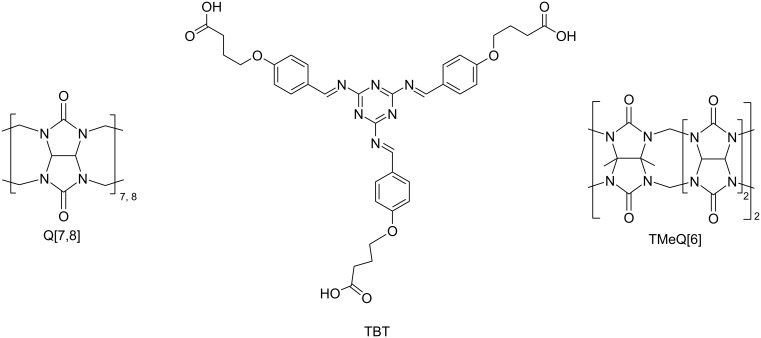
The Structures of Q[7], Q[8], TMeQ[6], and TBT.

Cucurbit[*n*]urils (Q[*n*]s, Scheme 1), a kind of supramolecular compound, are formed by polymerization of glycoluril, which contains rigid cavities for the study of host–guest chemistry and carbonyl groups for the study of coordination chemistry [16–20]. For the special methyl cucurbit[*n*]urils with abundant methyl groups, its high density of the electropositivity can be a perfect candidate for the study of outer-surface interaction of cucurbit[*n*]urils [21–24].

In this work, nitrogen-rich melamine is used as the center molecule to synthesize the Schiff base 2,4,6-tris(((4-(4-carboxybutyl)phenyl)methylene)amino)-1,3,5-triazine (TBT) through the Schiff base reaction and the nucleophilic reaction of haloalkane (Scheme 1 and Supporting Information File 1, Scheme S1) [25]. In addition to retaining the abundant coordination sites of Schiff bases, TBT is also modified with carbon chains of appropriate length for inducing host–guest interactions and with carboxyl groups for inducing the outer-surface interactions. Then three kinds of cucurbit[*n*]urils including tetramethylcucurbit[6]uril (TMeQ[6]), Q[7], and Q[8] are chosen as the hosts for TBT [26–31]. Due to the small cavity size and the higher density of the positive charge of TMeQ[6], TBT naturally interacts with the exposed methyl group on the outer surface through outer-surface interaction rather than entering its cavity. The cavity of Q[7] is very suitable for TBT, so Q[7] tightly binds with the branches of TBT through host–guest interactions. While Q[8] forms a supermolecule polymer with TBT due to its larger cavity. Since Q[7] and TBT form a host–guest complex, the carboxyl group at the end of TBT and the carbonyl group of the Q[7] still have a strong ability to coordinate with metals. Therefore, Q[7]-TBT is selected for the detection of silver ions (Ag^+^) [32–33].

## Results and Discussion

### The outer-surface interaction of TMeQ[6]-TBT

**Figure 1:**
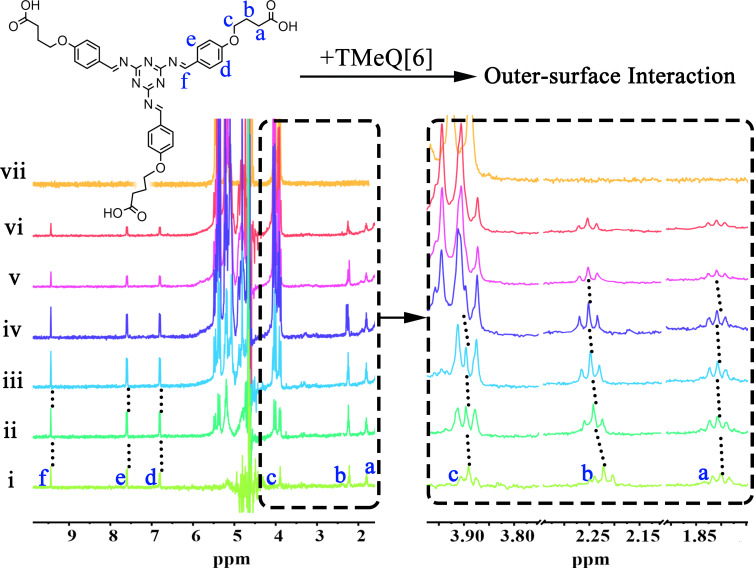
The ^1^H NMR titration of TBT (1 mM) with an increasing amount of TMeQ[6] from 0 (i), 0.1 (ii), 0.2 (iii), 0.6 (iv), 1.0 (v) to 1.4 equiv (vi), and the ^1^H NMR of free TMeQ[6] (vii) in D_2_O.

To investigate the outer-surface interaction between TMeQ[6] and TBT, ^1^H NMR titration is used. As shown in Figure 1, with the addition of TMeQ[6], the proton signals are shifted accordingly. For example, the signals of both H_a_, H_b_, and H_c_ are shifted downfield, while the signals of H_d_, H_e_, and H_f_ remain unchanged in the presence of TMeQ[6]. Therefore, it can be preliminarily inferred that the interaction between TMeQ[6] and TBT is mainly driven by the outer-surface interaction of the carboxylic carbon chain of TBT and the methyl or hydrogen of TMeQ[6] on its outer surface. In addition, when using UV–vis spectroscopy (Figure S5 and Supporting Information File 1, Figure S6) to investigate their interaction, it’s found that the presence of TMeQ[6] does not affect the absorbance of TBT, indicating that TMeQ[6] prefers to interact with TBT through outer-surface interaction than the host–guest interaction between the benzene ring or the melamine group of TBT.

### The host–guest interaction of Q[7]-TBT

**Figure 2:**
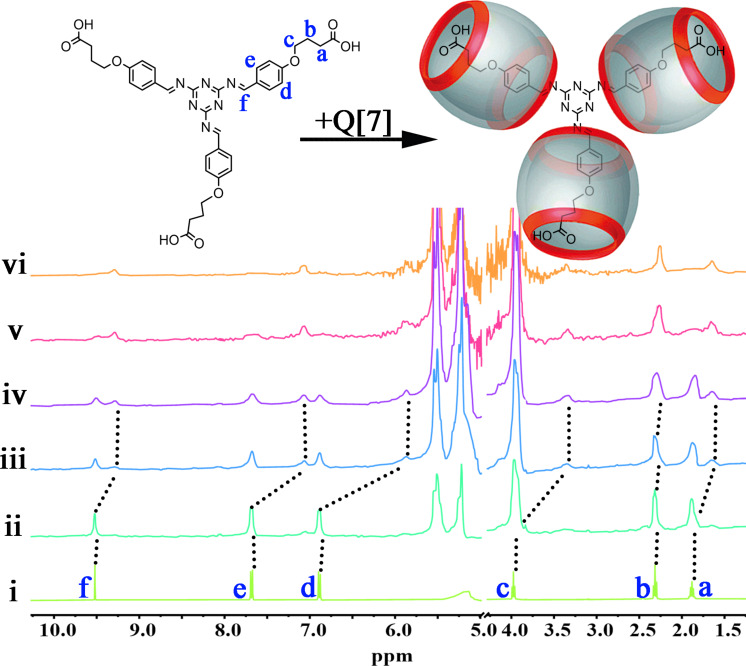
The ^1^H NMR titration of TBT (1 mM) with an increasing amount of Q[7] from 0 (i), 0.1 (ii), 0.5 (iii), 1.0 (iv), 2.0 (v) to 3.0 equiv (vi) in D_2_O.

Using the same method of ^1^H NMR titration as above. The interaction between Q[7] and TBT is studied and is proved to be different from that of TMeQ[6]. It is found that their interaction is the classical host–guest interaction instead of the outer-surface interaction because of the larger cavity of Q[7]. As shown in Figure 2, the proton signals of the entire TBT are shifted upfield with the increasing amount of Q[7]. For example, the signals of H_a_ shifted from δ = 1.88 ppm to 1.64 ppm, H_b_ from δ = 2.32 ppm to 2.25 ppm, H_c_ from δ = 3.97 ppm to 3.34 ppm, H_d_ from δ = 6.88 ppm to 5.87 ppm, H_e_ from δ = 7.68 ppm to 7.06 ppm and H_f_ from δ = 9.52 ppm to 9.29 ppm. Naturally, it can be inferred that Q[7] binds with the entire branches of TBT with strong host–guest interaction.

**Figure 3:**
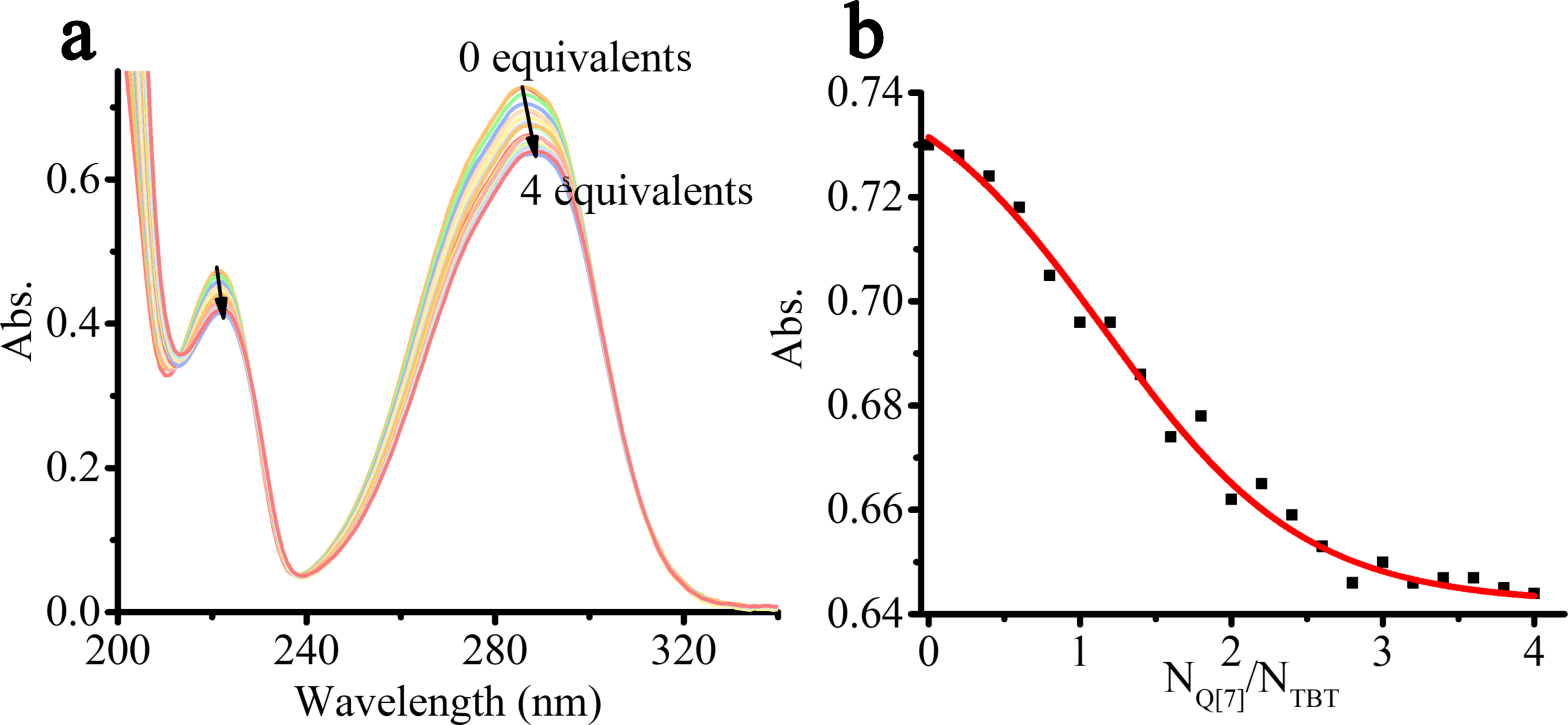
The UV–vis spectra (a) of TBT (20 μM) with an increasing amount of Q[7] from 0.0 to 4.0 equiv; the plots (b) of N_Q_[7]/N_TBT_ vs absorbance of TBT in water at λ = 286 nm.

In addition, we also used the UV–vis spectrum to verify the above inference and to further investigate their molar ratio in detail. Q[7] has a larger cavity than TMeQ[6], giving it the ability to bind TBT. Therefore, the absorbance of TBT gradually decreases and redshifts in the presence of Q[7] (Figure 3), which is mainly due to the π–π^*^ and n–π^*^ transition caused by the hydrophobic effect of the Q[7] cavity. Meanwhile, the absorbance of TBT is gradually approaching the saturation state, when the amount of Q[7] reaches 3 times that of TBT. Therefore, it can be inferred that Q[7] binds with the three “arms” of TBT at a molar ratio of 3:1 (N_Q_[7]/N_TBT_ = 3:1) and forms a host–guest complex Q[7]-TBT. In addition, the ITC experiment also strongly supports the above results, which data can be fitted to a very suitable curve using the model of sequential three site (Supporting Information File 1, Figure S7), and the corresponding binding ability (*K*_a_) is 1.422 × 10^6^ M^−1^.

### The host–guest interaction of Q[8]-TBT

**Figure 4:**
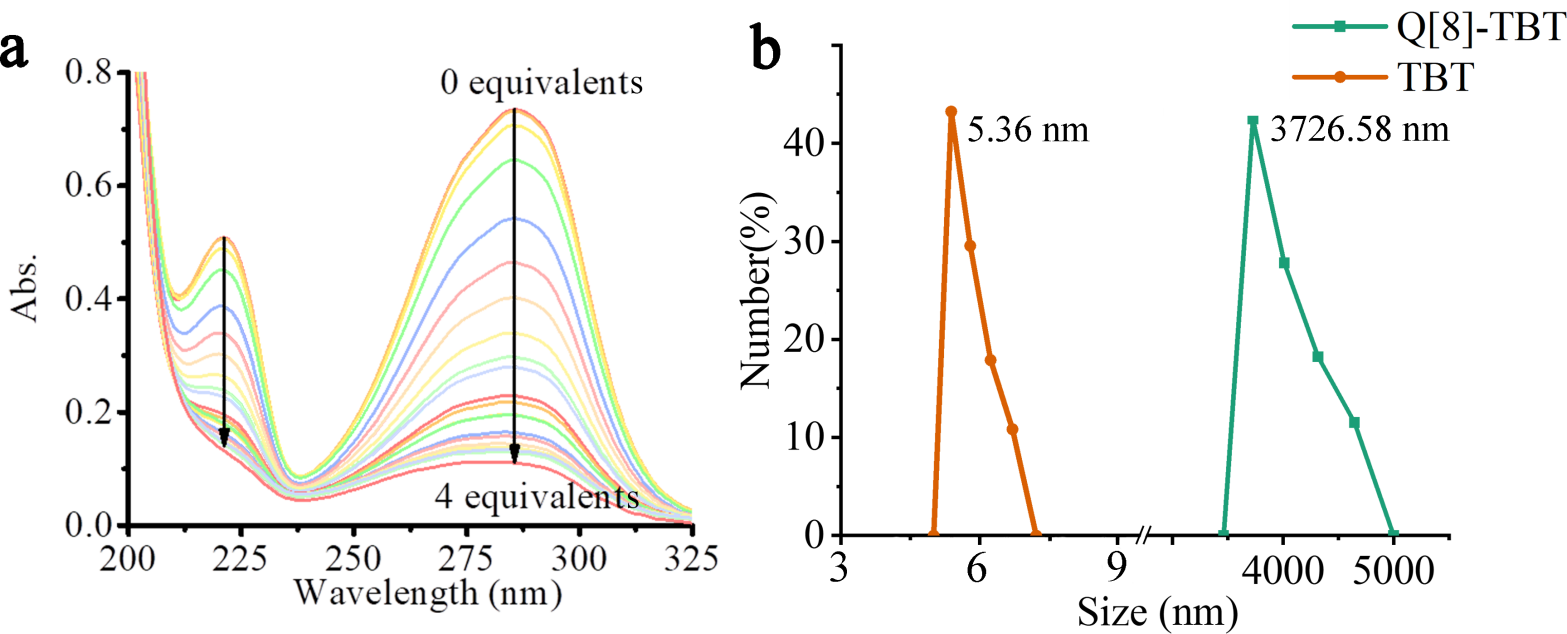
The UV–vis spectra (a) of TBT (20 μM) with an increasing amount of Q[8] from 0.0 to 4.0 equiv and the DLS of TBT (20 μM) and Q[8]-TBT (3:1, 20 μM).

Since Q[8] has a larger cavity than Q[7], it can bind the entire TBT molecule like Q[7]. However, in the ^1^H NMR titration experiment (Supporting Information File 1, Figure S8), it is found that upon the addition of Q[8], the chemical shift value of TBT did not change significantly. As the concentration of Q[8] continues to increase, the proton signals of TBT start to weaken, while the proton signals of Q[8] have not been detected during the whole experiment. In addition, the UV–vis spectrum in Figure 4a shows that the absorbance of TBT keeps on the decrease (ΔA = 0.623) without red shift or blue shift in the presence of Q[8]. Both of the above phenomena show that Q[8] interacted with the “arm” of TBT and produced precipitation due to aggregation, which is also the reason why the proton signals and absorbance of TBT in the ^1^H NMR and UV–vis spectra greatly declines. Then SEM and dynamic light scattering (DLS) are used for in-depth research of Q[8]-TBT complex. As shown in Figure 4b, compared with TBT (5.35 nm), the particle size of Q[8]-TBT is greatly increased to 3726.58 nm. At the same time, SEM (Supporting Information File 1, Figure S10) has also detected a large number of massive Q[8]-TBT complexes.

### Detection of Ag^+^ based on Q[7]-TBT

**Figure 5:**
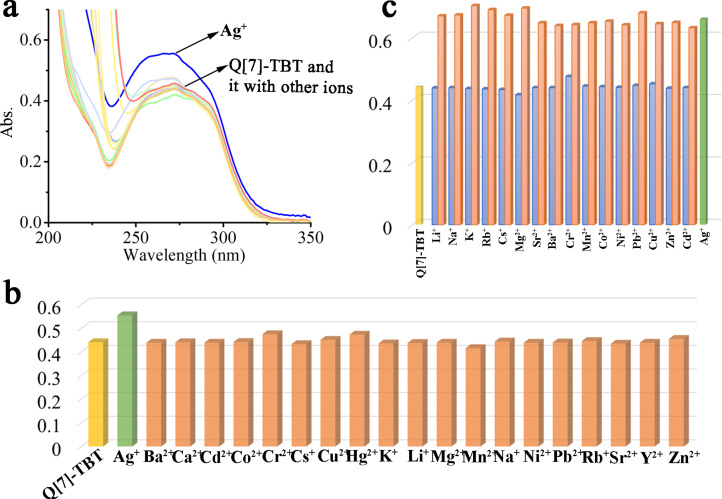
The UV–vis spectra (a) of Q[7]-TBT (3:1, 20 μM) affected by M*^n^*^+^ (50 equivalents); histogram of (b) Q[7]-TBT in the presence of M*^n^*^+^ and (c) the anti-interference experiment.

The guest molecule TBT contains three carboxyl groups and a wealth of lone-pair electrons, so it has a high coordination ability to metals. In this study, Q[7]-TBT was selected as a UV detector to detect common metals. As shown in Figure 5, the additional Ag^+^ increases the absorbance of the Q[7]-TBT complex from 0.441 to 0.555 at λ_max_ = 258 nm, while other metal ions do not increase or decrease the absorbance significantly at this wavelength, so Q[7]-TBT has a higher selectivity to Ag^+^ among metals. In addition, in the anti-interference experiment, Q[7]-TBT also has a good performance in the detection of silver ions in the presence of other common metals.

**Figure 6:**
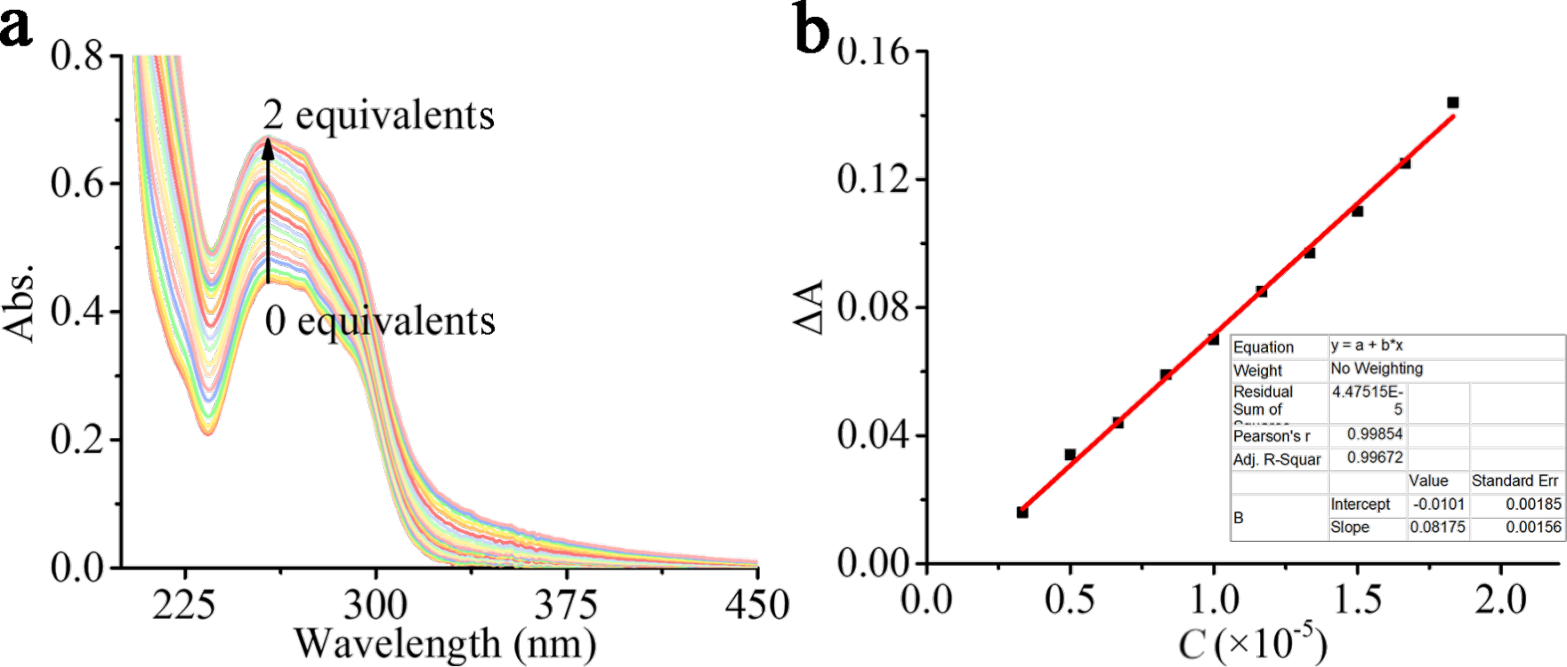
The UV–vis spectra (a) of Q[7]-TBT (20 μM) with an increasing amount of Ag^+^ from 0.0 to 2.0 equiv; DL plot (b) of Ag^+^.

To further explore the detection limit (DL) and detection mechanism of Q[7]-TBT towards Ag^+^, a UV–vis titration experiment was carried out. As shown in Figure 6, with the continuous addition of Ag^+^, the absorbance of Q[7]-TBT continues to increase at λ_max_ = 258 nm, which is caused by the n–π^*^ transition of Q[7]-TBT. Therefore, it can be further inferred that Ag^+^ mainly binds with the carboxyl group of TBT. In addition, the DL is calculated to be 3.91 × 10^−6^ M, which perfectly fits with the formula y = −0.01 + 0.0182x, *R*^2^ = 0.997.

## Conclusion

Three different complexes, TMeQ[6]-TBT, Q[7]-TBT, and Q[8]-TBT, are constructed from three different cucurbiturils with the same molecule TBT. Due to the subtle attribute gap between cucurbiturils, the TMeQ[6]-TBT complex is driven by outer-surface interaction, the Q[7]-TBT and Q[8]-TBT complexes are formed by host–guest interactions. Finally, Q[7]-TBT is selected as a UV detector for the detection of silver ions (Ag^+^). This work fully demonstrates the charm of the rigid cavity of cucurbiturils. Different cucurbiturils can selectively bind guest molecules differently according to their characteristics. In addition, the Q[7]-TBT complex constructed in this paper is further applied to metal detection due to the strong coordination ability of Schiff base, providing a theoretical study for the Q[*n*]-Schiff base complex.

## Experimental

**Scheme 2:**
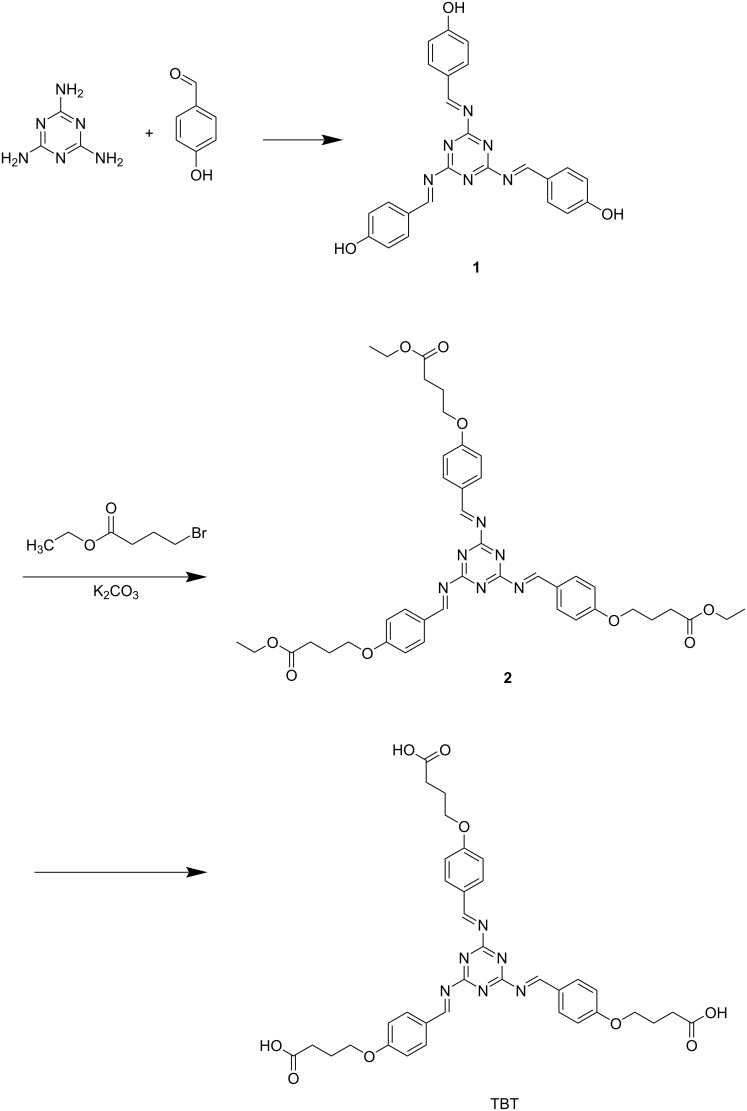
The synthesis of TBT.

**The synthesis of 1:** Compound **1** is synthesized according to the literature (Scheme 2) [34]. Melamine (10 mmol, 1.26 g) was suspended in benzene (20 mL) and a suspension of 4-hydroxybenzaldehyde (30 mmol, 3.66 g) in benzene (30 mL) was added under stirring, then reflux overnight. The pink powder was formed and washed with warm water to get the pure compound **1**. ^1^H NMR (DMSO-*d*_6_) δ 12.1 (s, 3H), 9.76 (s, 3H), 7.80 (d, 6H), 7.08 (d, 6H), 4.06 (t, 6H), 2.40 (t, 6H), 1.93 (m, 6H) ppm.

**The synthesis of 2:** K_2_CO_3_ (0.138 g, 1.0 mmol) and compound **1** (0.438 g, 1.0 mmol) were dissolved in acetonitrile (80 mL) and refluxed for 3 h. Ethyl 4-bromobutyrate (0.918 g, 3 mmol) was added and refluxed for a day. The solvent was removed, and then separated by column chromatography (EA/PE) to get the pure compound **2**. ^1^H NMR (DMSO-*d*_6_) δ 9.82 (s, 3H), 7.82 (d, 6H), 7.07 (d, 6H), 7.08 (d, 6H), 4.04 (m, 14H), 1.95 (m, 9H), 1.13 (t, 12H) ppm.

**The synthesis of TBT:** Compound **2** (0.780 g, 1.0 mmol)) and NaOH (0.27 g, 6.75 mmol) were combined in a 1:1 solution of acetonitrile and water (20 mL) and refluxed for 4 h. The mixture was concentrated under vacuum and then acidified by HCl to pH 2 to precipitate a white solid. ^1^H NMR (D_2_O) δ 9.52 (s, 3H), 7.68 (d, 6H), 6.89 (d, 6H), 3.98 (t, 6H), 1.90 (t, 6H), 1.92 (m, 6H) ppm.

## Supporting Information

File 1 Experimental and analytical data.
