# Supplementary material for: Study on the interactions between melamine-cored Schiff bases with cucurbit[n]urils of different sizes and its application in detecting silver ions
Source: Beilstein J Org Chem. 2021 Dec 17;17:2950–8. doi: 10.3762/bjoc.17.204 (PMC8685555; doi:10.3762/bjoc.17.204)
Supplement: File 1 — Experimental and analytical data. [file Beilstein_J_Org_Chem-17-2950-s001.pdf]

BEILSTEIN JOURNAL OF ORGANIC CHEMISTRY

Supporting Information

for

Study on the interactions between melamine-cored Schiff bases with cucurbit[*n*]urils of different sizes and its application in detecting silver ions

Jun-Xian Gou, Yang Luo, Xi-Nan Yang, Wei Zhang, Ji-Hong Lu, Zhu Tao and Xin Xiao

Beilstein J. Org. Chem. **2021,** *17,* 2950–2958. doi:10.3762/bjoc.17.204

Experimental and analytical data

License and Terms: This is a supporting information file under the terms of the Creative Commons Attribution License (https://creativecommons.org/licenses/by/4.0). Please note that the reuse, redistribution and reproduction in particular requires that the author(s) and source are credited and that individual graphics may be subject to special legal provisions.
The license is subject to the *Beilstein Journal of Organic Chemistry* terms and conditions: (https://www.beilstein-journals.org/bjoc/terms)

Content

Materials S3

Measurement of UV–vis spectra S3

^{1}H NMR measurements S3

Isothermal titration calorimetry (ITC) measurements S3

Measurement of the limit of detection S4

The experimental method of anti-interference experiment S4

Scheme S1 The synthesis routes of TBT S5

Figure S1 The ^{1}H NMR of **1** S5

Figure S2 COSY of **1** S6

Figure S3 The ^{1}H NMR of **2** S6

Figure S4 The ^{1}H NMR and COSY of **TBT** S7

Figure S5 The UV–vis of TBT (20 μM) towards TMeQ[6] S8

Figure S6 The plot of $N_{TMeQ[6]}/N_{TBT}$ *vs.* abs. at λ= 286nm. S8

Figure S7 ITC of Q[7] (0.1 M) towards TBT(0.005 M). S9

Table S1 Date of ITC S9

Figure S8 The ^{1}H NMR titration of TBT (1 mM) with an increasing amount of Q[8] from 0, 0.5, 1.0 to 2.0 from bottom to top, and free Q[8] (top) in D_2O. S10

Figure S9 The plot of $N_{Q[8]}/N_{TBT}$ vs abs. at λ= 286nm. S10

Figure S10 The SEM of Q[8]-TBT (1 mM). S11

Figure S11 The plot of $N_{Ag+}/N_{Q[7]\text{-}TBT}$ *vs.* abs. at λ = 286nm. S11

Materials

Q[7], Q[7] and TMeQ[6] were synthesized according to a procedure developed previously in our laboratory. Melamine, 4-hydroxybenzaldehyde, K_2CO_3 and ethyl 4-bromobutyrate were obtained from Sigma-Aldrich (Shanghai, China) and corresponding perchlorate salts were obtained from Aladdin (Shanghai, China). All reagents were of analytical reagent grade and were used without further purification. Doubly-distilled water was used throughout.

Measurement of UV–vis spectra

All UV-visible spectra were recorded on an Agilent 8453 spectrophotometer (Agilent Technologies, Santa Clara, CA, USA), from solutions in 1 cm quartz cells.

Aqueous solutions of TBT (20 μM) were prepared by diluting the stock solutions, and an increasing concentration (0–80 μM) of Q[*n*] solution was added to free TBT to obtain plot of $N_{Q[7]}/N_{TBT}$ vs absorbance of TBT.

For the part of detection of Ag^+: Aqueous solutions of the Q[7]-TBT complex (3:1, 20 μM) were prepared for characterization by UV–vis spectra. Then known quantities of metal ion solutions were added to Q[7]-TBT to obtain the corresponding plot.

^{1}H NMR measurements

The ^{1}H NMR spectra were recorded at 25 °C on a Jeol JNM-ECZ400s spectrometer. D_2O was used as a field-frequency lock and the observed chemical shifts are reported in parts per million (ppm) relative to that for the internaltetramethylsilane (TMS) standard (0.0 ppm).

Isothermal titration calorimetry (ITC) measurements

Thermodynamic parameters and binding constants (K) for the Q[7]-TBT were determined by ITC using a Nano ITC instrument (TA, USA). All solutions were prepared in doubly-distilled water and degassed prior to titration experiments. The heat evolved was recorded at 298.15 K. The heat of dilution was corrected by injecting guest solution (free guest) into an aqueous solution and subtracting the

values from the corresponding values obtained for the host–guest titration. Computer simulations (curve fitting) were performed using Nano ITC analytical software. For Q[7]-TBT, the concentration of Q[7] in the sample cell (1.3 mL) was 1×10^{-4} mol/L. A typical ITC titration was carried out by titrating the TBT solution (1×10^{-3} mol/L, 10 μL aliquots, at 250 s intervals) into Q[7] solution.

Measurement of the limit of detection

The calculation method used for the limit of detection (DL) was based on the standard derivation of ten measurements in the absence of the guest molecule (σ) and the slope of the linear calibration curve (K) according to the formula: DL =3σ/K.

The experimental method of anti-interference experiment

Other interfering metals (50 equivalents) were separately added to the solution of Q[7]-TBT (3:1, 20 μM), then their absorption values were recorded. Subsequently, the detected ion (Ag^{+}) is immediately added into the above mix solution of Q[7]-TBT and interfering metals, then recorded the absorption value once again.

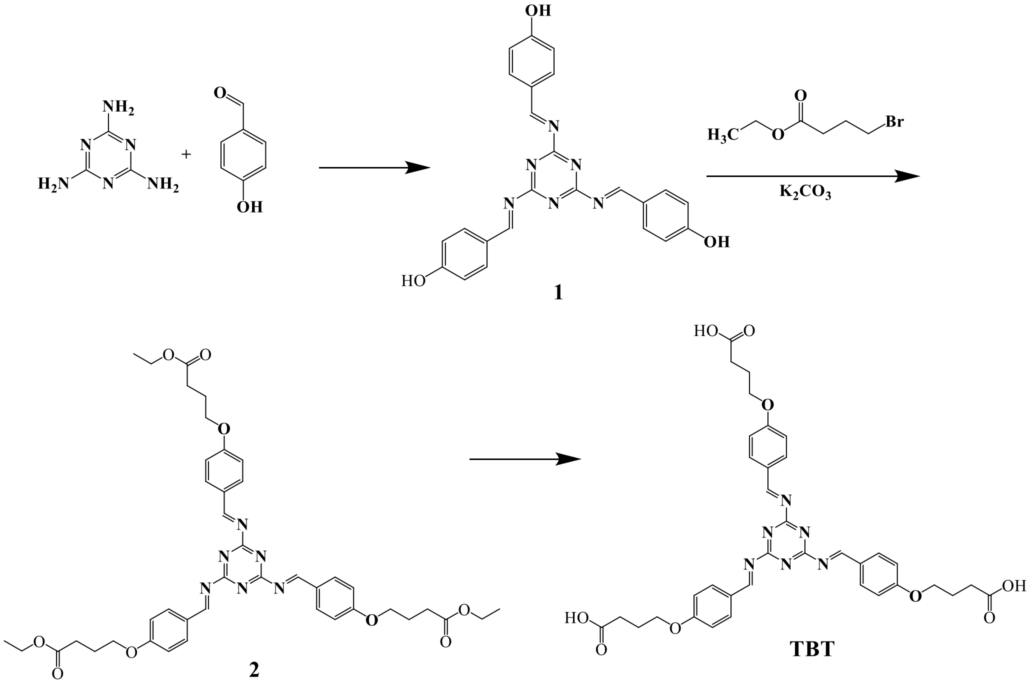

Scheme S1: The synthesis routes of TBT.

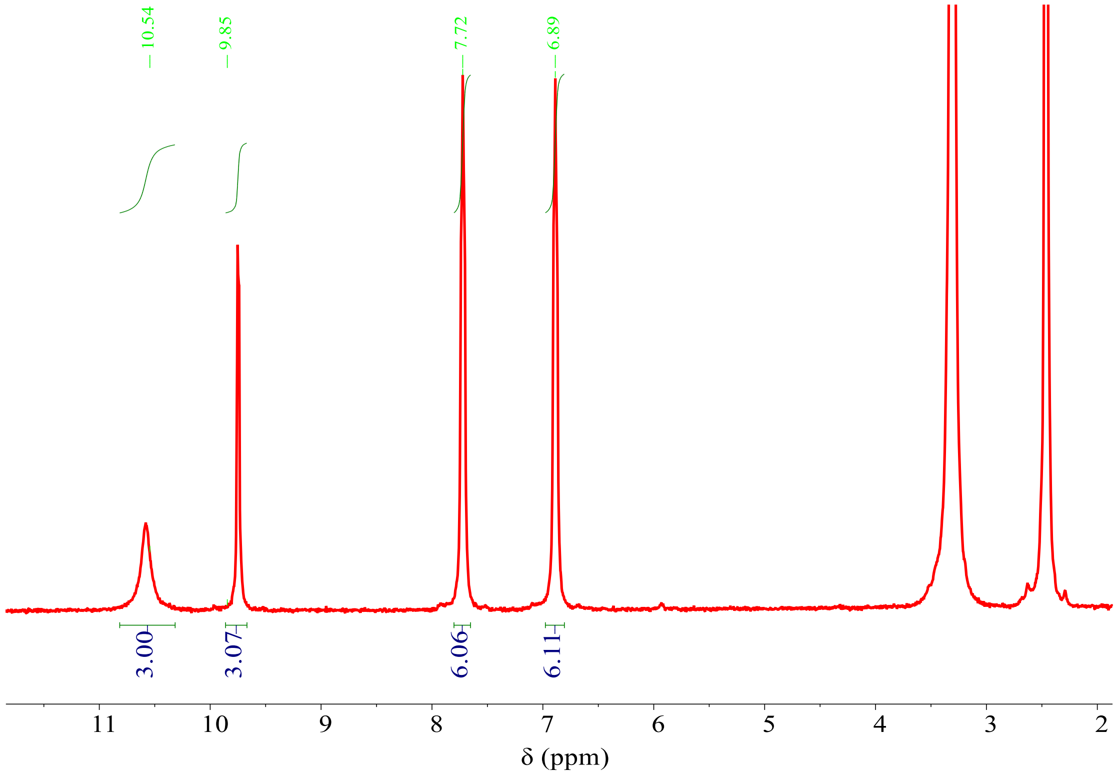

Figure S1: The ^{1}H NMR of **1**.

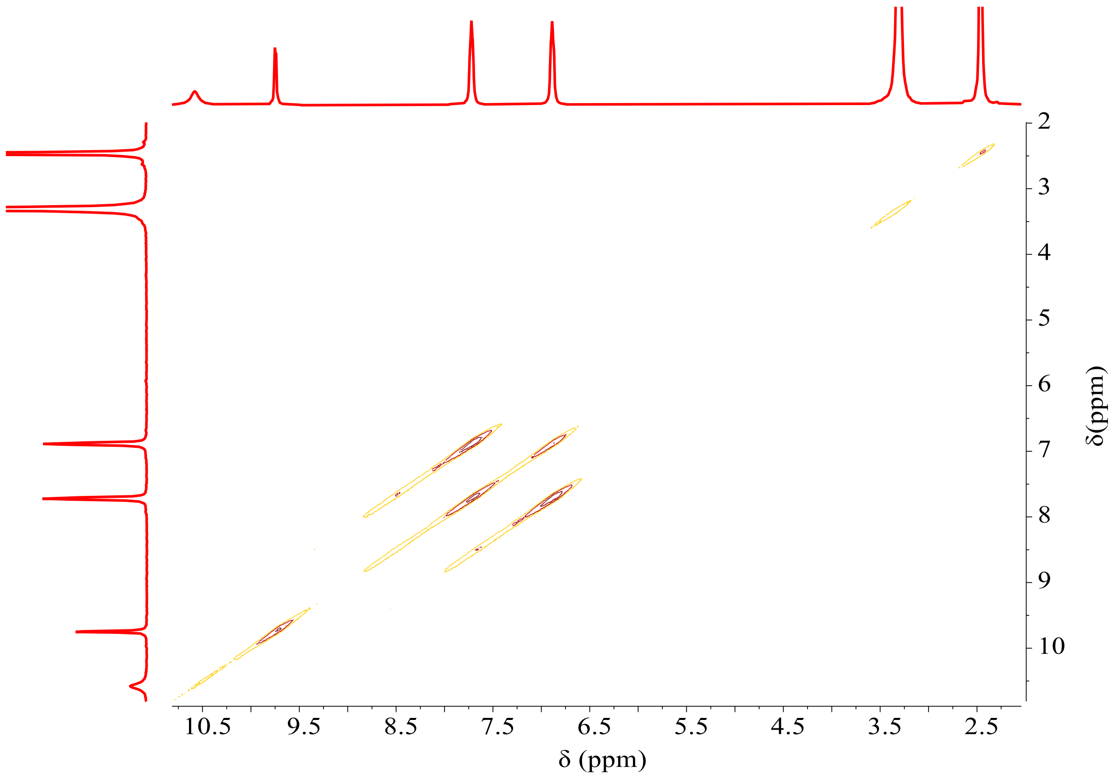

Figure S2: COSY of **1**.

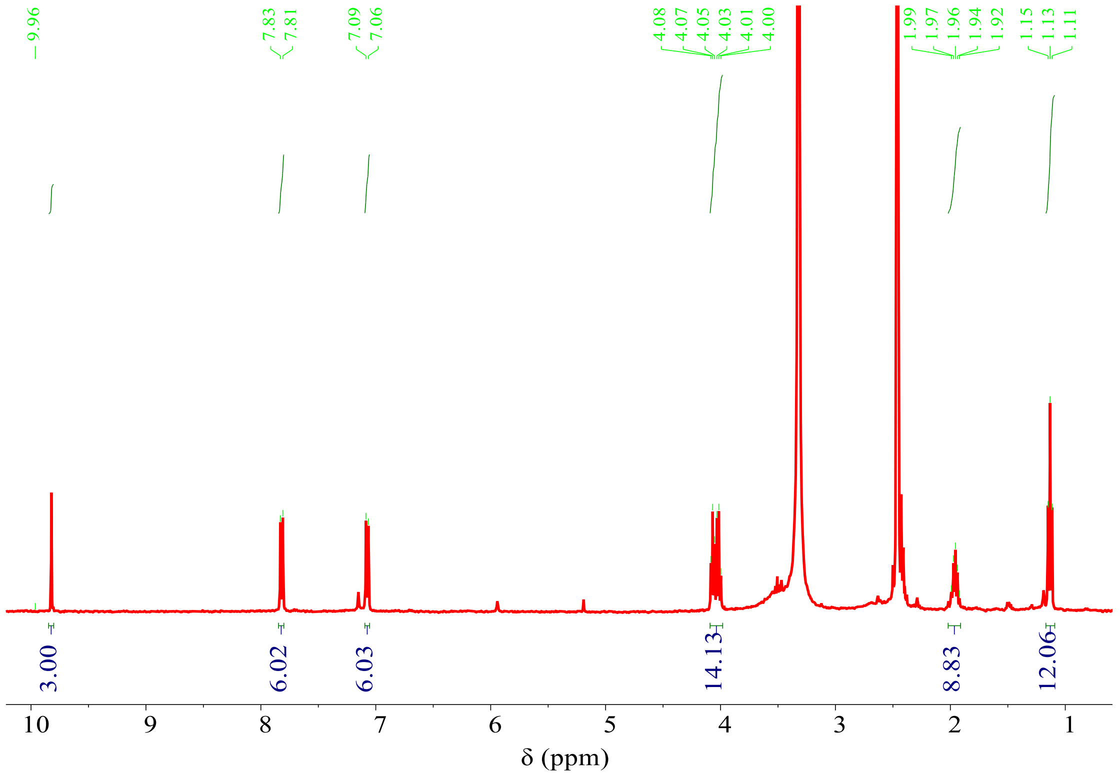

Figure S3: The ^{1}H NMR of **2**.

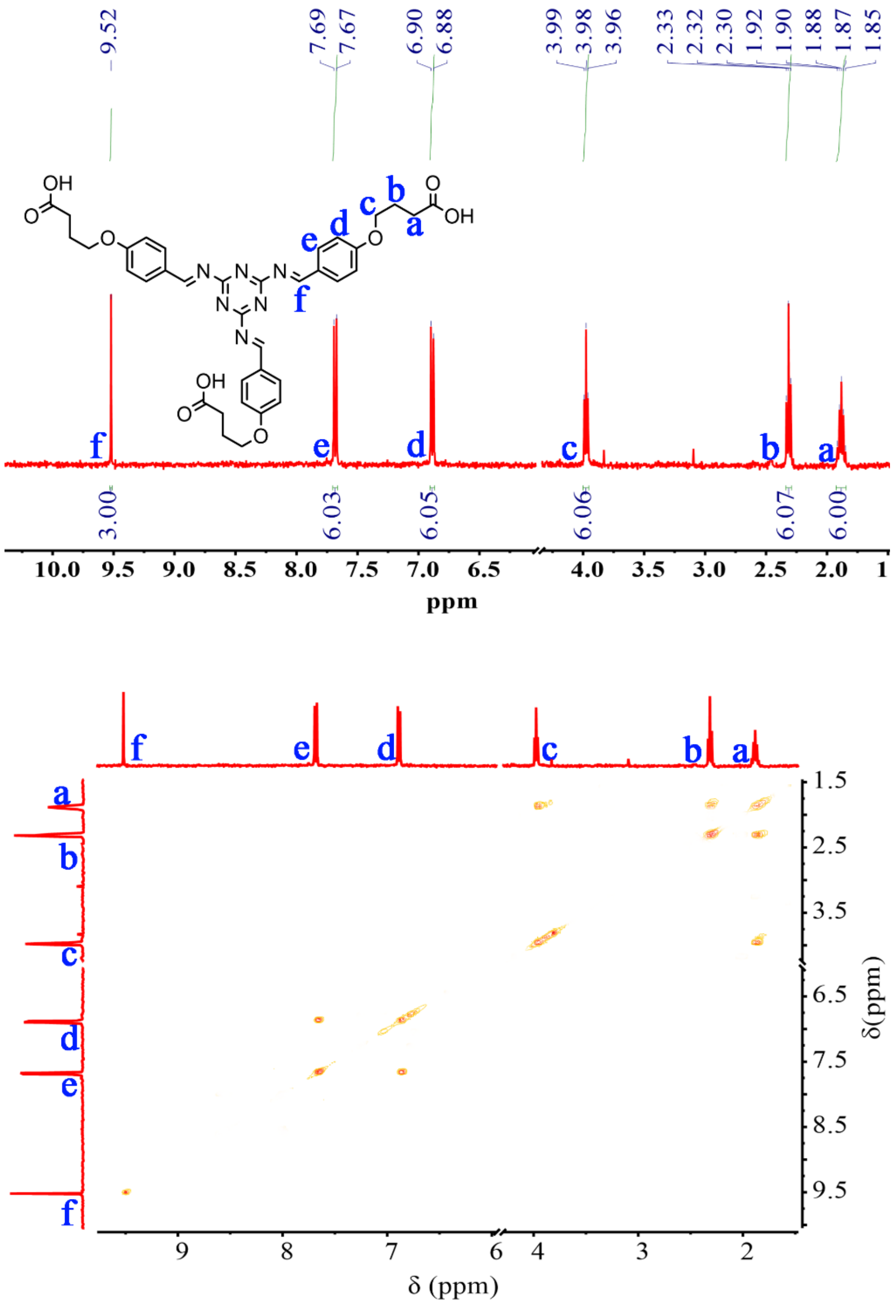

Figure S4: The ^{1}H NMR and COSY of **TBT**.

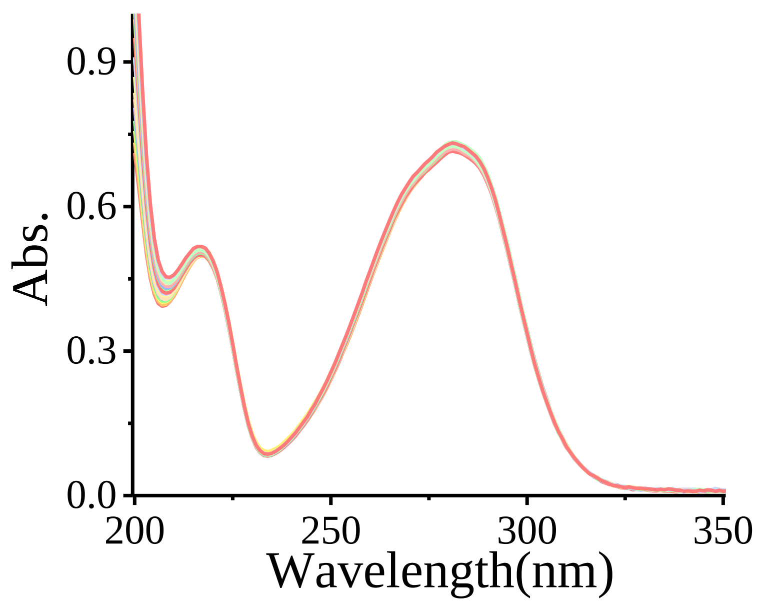

Figure S5: The UV–vis of TBT (20 μM) towards TMeQ[6].

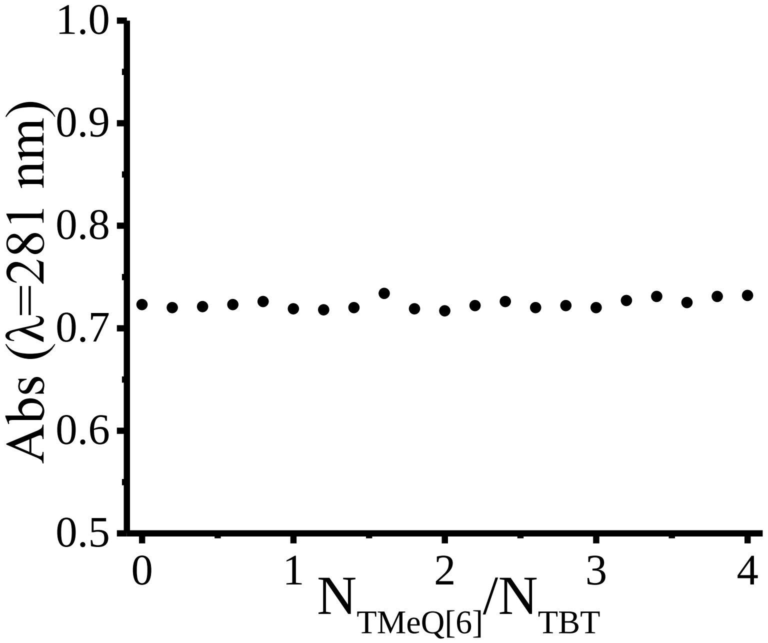

Figure S6: The plot of $N_{TMeQ[6]}/N_{TBT}$ vs abs. at λ = 286nm.

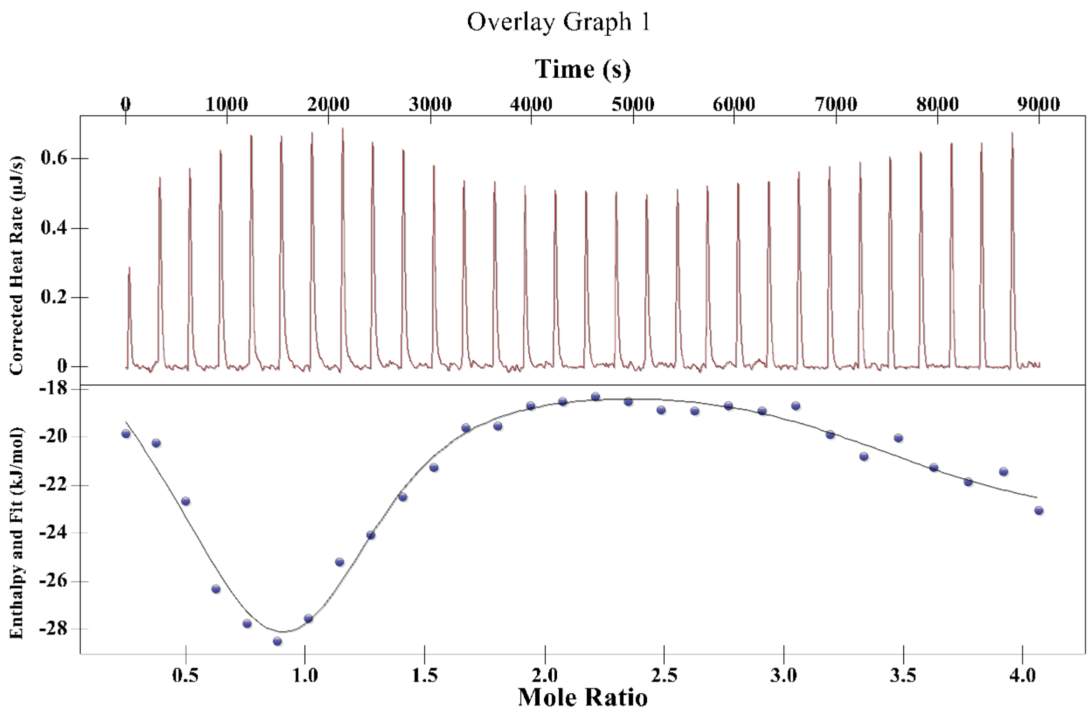

Figure S7: ITC of Q[7] (0.1 M) towards TBT(0.005 M).

Table S1: Data of ITC

K_{a1} (M^{-1})	K_{a2}(M^{-1})	K_{a3}(M^{-1})
1.422×10^6	2022	21.55
ΔH_1 (KJ/mol)	ΔH_2 (KJ/mol)	ΔH_3 (KJ/mol)
62.72	−3918	−1783
ΔS_1 (J/mol·K)	ΔS_2 (J/mol·K)	ΔS_3 (J/mol·K)
328.2	-1.308×10^4	-5.954×10^3

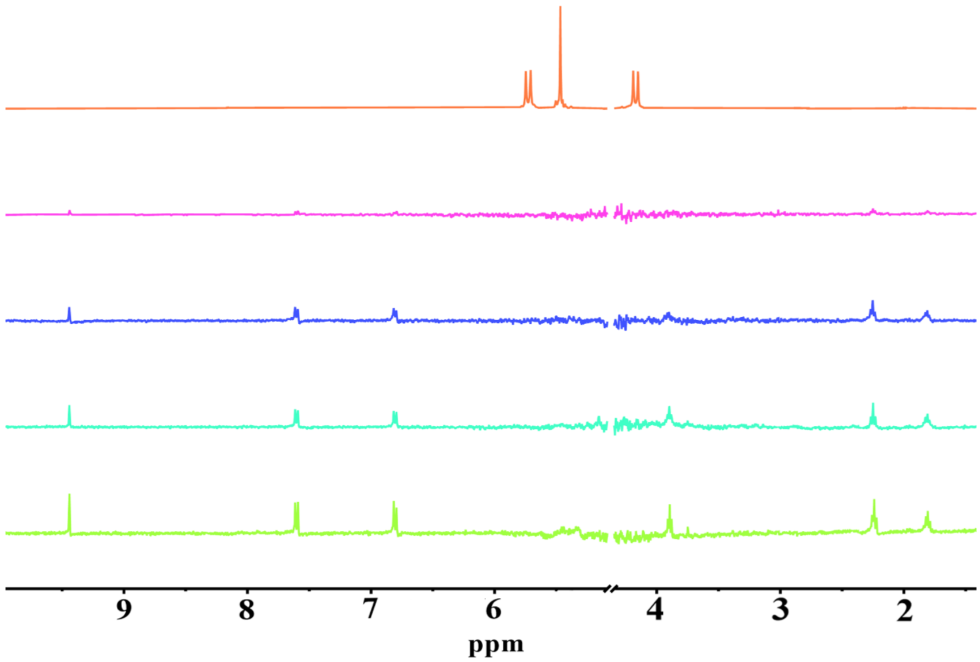

Figure S8: The ^{1}H NMR titration of TBT (1 mM) with an increasing amount of Q[8] from 0, 0.5, 1.0 to 2.0 from bottom to top, and free Q[8] (top) in D_2O.

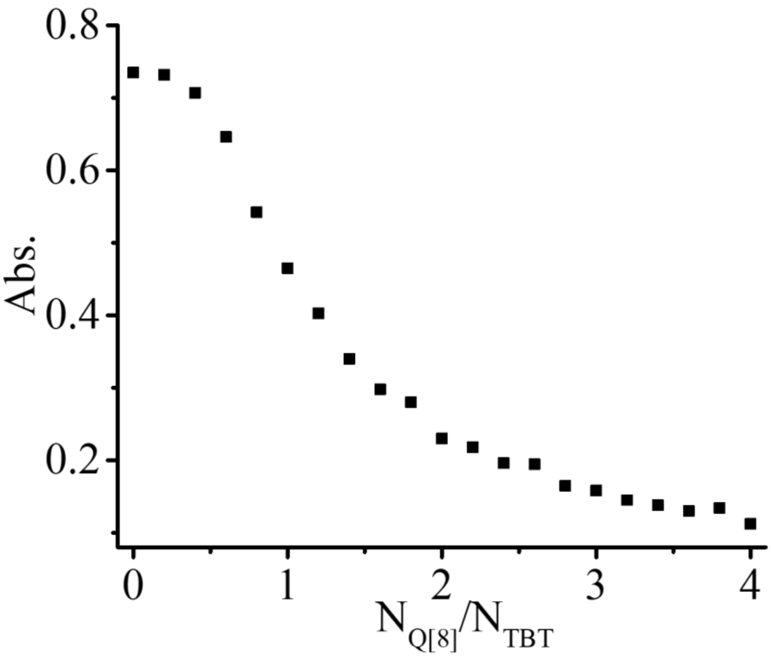

Figure S9: The plot of $N_{Q[8]}/N_{TBT}$ vs abs. at λ = 286nm.

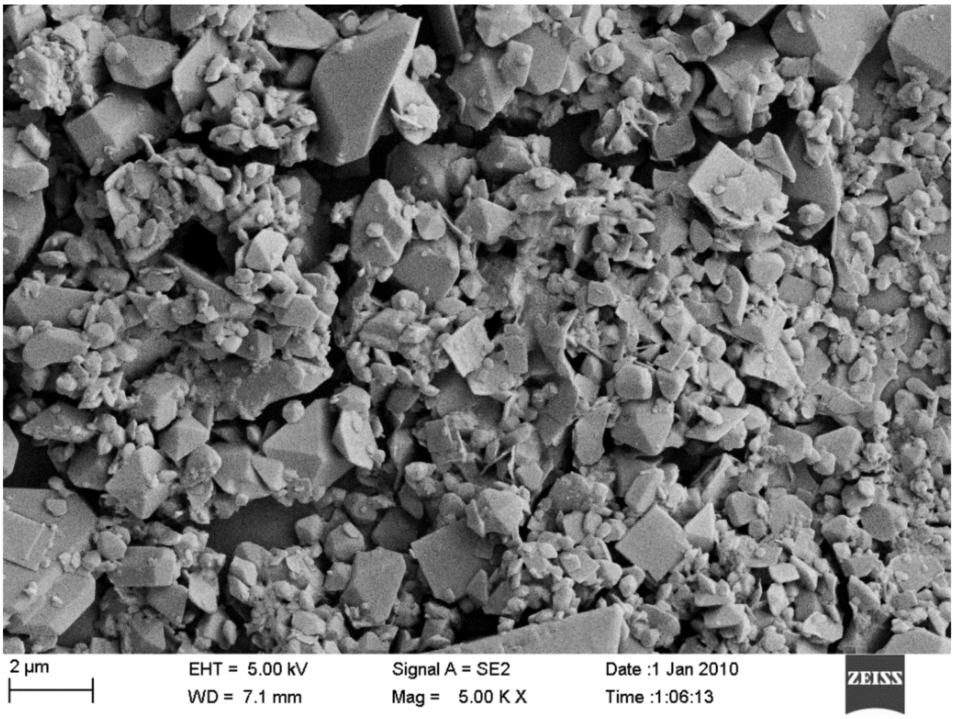

Figure S10: The SEM of Q[8]-TBT (1 mM).

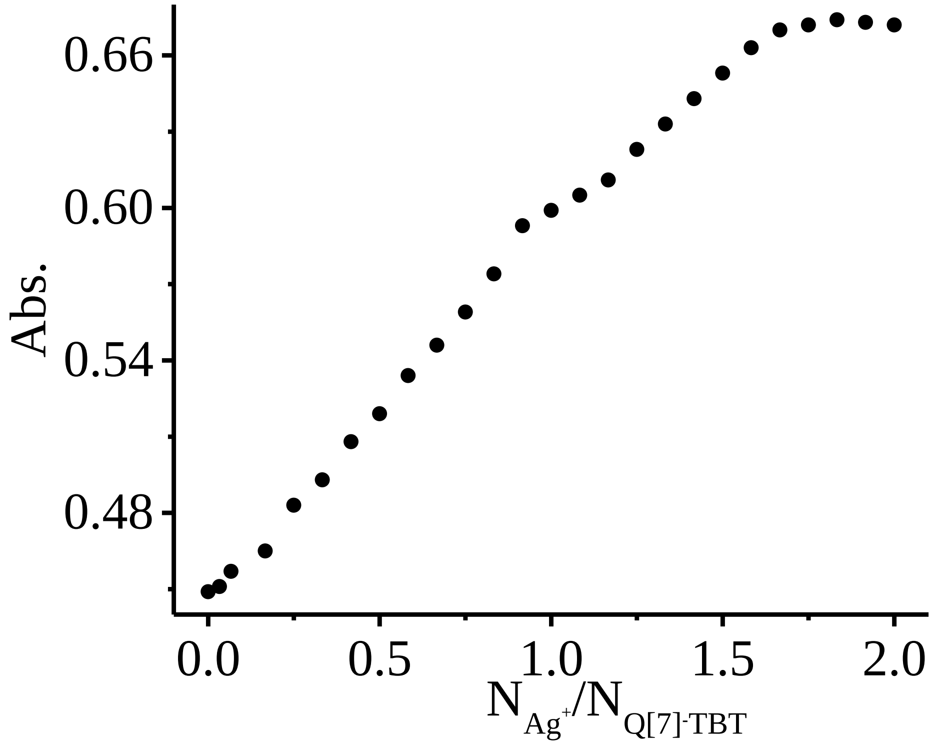

Figure S11: The plot of $N_{Ag+}/N_{Q[7]-TBT}$ vs abs. at λ = 286nm.
